# Publications on Anatomy

**Published:** 1838-04-01

**Authors:** 


### PUBLICATIONS ON ANATOMY.

I. Observations on the Structure and Functions of the Spinal Cord. By *R. A. Grainger*, Lecturer on Anatomy and Physiology. Octavo, pp. 159. London, 1837.

II. Observations on the Ganglionic Enlargement of the Pneumo-Gastric Nerve; the probable Function of that Ganglion; and the Position which it occupies in the Human Subject and in several of the Lower Animals. By *Mr. Edward Cock.*

[Guy's Hospital Reports, Oct. 1837.]

III. On the Distribution and probable Function of the superior and recurrent Laryngeal Nerves. By Mr. *John Hilton.*—Ibid.

IV. Description of the Sacculus or Pouch in the Human Larynx. By Mr. *John Hilton.*—Ibid.

In the last number of this Journal we presented our readers with an account of the major portion of Mr. Grainger's work upon the spinal cord—a work which fully merits attention both from them and from us.* Of the seven chapters of which it is composed, we gave a copious notice of four, and deferred to the present number an analysis of the remainder.

We shall say nothing at this moment of the paper of Mr. Cock, nor of those of Mr. Hilton, but devote to them, and to the subject of which they treat, the consideration they deserve anon.

### I. Anatomy of the Spinal Cord.

We have already shewn that the principal interest of Mr. Grainger's volume, attaches to the anatomical development of the excito-motory system attempted in it. That system has attracted and must still attract so much attention, that the facts advanced in its support, as well as the reasoning employed for the same purpose, should be familiar to the bulk of the profession.

In our last number we arrived at the fifth chapter of Mr. Grainger's work. It is devoted to the exhibition of the "General Results" of the enquiries into the anatomy and physiology of the cord, contained in the preceding chapters. It would be useless, we conceive, to recapitulate the details of those inquiries, for which we may at once refer our readers to the article in our last number. We shall therefore proceed without further delay to those General Results to which we have alluded.

* Vide No. 55, pp. 116, et seq.

A. GENERAL RESULTS.

Mr. Grainger, in opening this chapter, again takes occasion to deplore the confusion which prevails in our ideas of the operations of the nervous system, and to hail the excito-motory theory as the light which dispels, or, at the least, diminishes the gloom. As he observes, the great object is to determine the true seat of sensation and volition. Whether he has done that or not, we shall not, at present, pause to enquire. Be this as it may, Mr. Grainger believes that the following axioms appear to be susceptible of proof.

"1. That the source of all power, in the nervous system, is the grey matter.

"2. That the white fibres are merely conductors.

"3. That there exist, in the nervous system, two great divisions—A, the true cerebral; consisting of the hemispheres of the cerebrum, and the lobes of the cerebellum; of the true sensiferous, and the true volition fibres of the cranio-spinal nerves;—B, the true spinal; comprising the grey matter of the spinal cord, and the incident and reflex fibres of the cranio-spinal nerves.

4. That there is no special order of respiratory nerves.

5. That there is but one kind of sensibility possessed by animals; that, namely, which is perceived by the mind.

6. That this sensibility is, in the higher animals, the invariable and inseparable property of the cerebral hemispheres, inclusive of the lobes of the cerebellum; and, in the lower animals, of that part of the nervous system which can be shown to be, in office, the true analogue of the brain.

7. That volition is the inseparable attribute of the cerebral hemispheres, and lobes of the cerebellum.

8. That the spinal cord, in every class of the animal kingdom in which it exists; and the analogous part, in those animals in which, in consequence of variety of shape or other circumstances, this cord cannot be detected in the usual form, is the inherent seat of a property, totally distinct from sensation and volition, called the reflex power.

9. That the reflex power is never exercised, without the excitement caused by the application of a physical agent to the external and internal surfaces of the body.

10. That contractility is the special property of the muscular fibre.

11. That contractility has no necessary connexion with sensibility.

12. That contractility cannot be exercised without the application of a stimulus.

13. That this stimulus consists of—A, volition; B, the reflex power of the spinal cord; and, *perhaps*, C, of a direct application of a physical agent to the muscular fibre itself." 120.

On these axioms, Mr. Grainger comments individually.

1. In reference to the first and second, Mr. Grainger remarks, *that* the mode of connexion between the grey and fibrous substances of the nervous system being apparently uniform—*that*, the former being highly vascular—*that* innumerable torrents of blood incessantly rushing through the part where the two substances come together, and suspension of the power of the nervous system resulting from the total interruption of this current, even for a few seconds—the *true idea* of the nervous substance is, consequently, that it consists of white fibres, separated from each other by portions of the grey matter, and of incessant currents of blood rushing through the intervals. These considerations, joined to the facts which have been already adduced, leave no doubt as to the exact seat of the nervous power. However plausible, we must remember that this is still theory, and we must not be too satisfied with our knowledge of the properties of the grey and white portions of the nervous system, to induce us to relax in our enquiries, or to repose great confidence on what we appear to have ascertained.

3. We need not resume the discussion of the question regarding the true seat of the excito-motory actions. That question was considered as fully as the case admits at present, in our last number, and it would be useless re-opening it. But the following observations relating to another and a very interesting point, demand a notice. They refer to the respective functions of the anterior and posterior roots of the spinal nerves.

"In the course of the experiments related in the previous chapter, it was often observed that on touching the *posterior* column of the spinal cord, motion was produced. This fact, which has also been remarked by several experimentalists, constitutes the principal ground-work of the objections urged against the doctrine of Bell. Although I am, myself, perfectly convinced of the exactness of the deductions drawn by this distinguished physiologist from his observations and experiments, and although they have been generally received by the first authorities in Europe; yet, whilst such men as Meckel, Rudolphi, and Weber, still regard these doctrines as being conjectural, the question as to the functions of the anterior and posterior roots of the spinal nerves cannot be considered as determined.

On irritating the posterior part of the spinal cord, it is, as I have stated, often observed that motion is excited. In connexion with this subject, Mr. Barron, with great justness remarked, that inasmuch as irritation of the incident nerves, where they terminate in the skin, excites muscular action, it must necessarily happen that when these nerves are irritated where they enter the spinal cord, a similar result will be produced. It is certain when the posterior surface of the cord is irritated, that sometimes the incident nerves will be touched, and whenever this occurs muscular action must take place. In this manner it is easy to reconcile those conflicting results, which have hitherto constituted an unexplained anomaly in the theory of Bell and Magendie." 124.

This is certainly an ingenious mode of explaining the phenomenon referred to. Whether it be the true one, or whether the whole subject is not still open to investigation, are points on which we shall not touch.

4. Dr. Hall has pointed out, what most physiologists had made up their minds to, that Sir C. Bell's theory of the "respiratory system" of nerves, however captivating, is fallacious. Dr. Hall modifies and extends that theory, so as to make it a piece of his own more comprehensive one, and probably his view is the correct one. He says—"I perfectly agree with Sir Charles Bell in the opinion that the respiratory is entirely distinct from the other subdivisions of the nervous system; but I venture to differ from him in viewing the respiratory as but *a part* of a more extensive system—as an *excited* and not a *spontaneous* function—as *originating*, when the cerebrum is removed, in the pneumo-gastric *as its excitor*, and not in the medulla oblongata."

Mr. Grainger adds that the reason of the great importance of the medulla oblongata, is because the pneumo-gastric, the "excitor" nerve of respiration is attached to it.

5. We are induced to extract the whole of the succeeding passage.

"If it had not happened," says our author, "that physiological writers, in consequence of the difficulties which are involved in all the existing theories, had admitted two kinds of sensibility, animal and organic, one of which, it was supposed, was, and the other was not perceived by the mind, it would have been surprising that such a distinction should ever have been attempted. If we free ourselves for an instant of all these confused notions, it becomes evident that there is but one kind of sensibility,—for the very term sensation implies something of which the mind is conscious; thus, for example, if I touch a piece of wood with the finger, a certain effect is produced on the ends of the sentient nerves, which is called an impression; and this impression, when it has been transmitted to the brain, is by the agency of that organ perceived, and then, but not till then, sensation is produced. The blood makes an impression on the internal surface of the heart, but as this impression is not under ordinary circumstances transmitted to the brain, it is not perceived, and, consequently, sensation is not produced. By this illustration, it is not meant to be denied that the heart, the stomach, the intestines, &c., are endowed with a capability of receiving the impressions of the blood, the food, or any other physical agent; or that these impressions do not excite the muscular action of these organs. All I mean to express is, that these impressions made on the heart, stomach, and so forth, do not cause sensation—they excite motion but not feeling. If the term sensibility be employed to indicate simply the power which the nervous system, viewed in a collective manner, has of receiving every kind of impression, whether attended with perception or not, there is only an inexactness of phraseology, and not a positive error; but inasmuch as the opinions of many persons are influenced more by the words than by the facts they are intended to convey, it is indispensable to banish from physiology, a laxity of expression which has been productive of incalculable confusion. The terms sensibility and sensation which are generally used synonymously, although in fact the former is the cause, and the latter only the effect, ought in future to be restricted to sensation attended with consciousness, which, indeed, with the exception of physiological writers, is the meaning universally attached to the expression. As to that power which the true spinal cord possesses of receiving impressions which are not attended by consciousness, but which produce motion, it has no necessary connexion with sensation, and, therefore, should be distinguished by a separate name; and although the word is not free from objection, yet, as it corresponds with the name given to the phenomena which result from the property in question (the *excito-motory.)* The latter may be termed *excitability.* The source of all this fallacy has been clearly pointed out by Dr. Hall." 127.

We quite agree with Mr. Grainger on the inconvenience of the present nomenclature. Others have also pointed out that inconvenience, and endeavoured, but with indifferent success, to remedy it. Among these we may cite Dr. Bostock, who proposes a remedy which aggravates the distemper.

It would certainly be better to limit the terms sensibility and sensation to that power of receiving impressions, and to the actual reception of them, accompanied with consciousness. Physiological, philosophical, and common language, would then harmonize. The organic sensibility of Bichât is certainly a paradox in terms—implying as it does a sensibility without sensation. "Excitability" is in this sense less objectionable, and less liable to give rise to confused ideas. The criticism of Mr. Grainger, it must be seen, is only a verbal one, for no one, possessed of any information, conceived that the organic sensibility was any thing else than what is now proposed to be called "excitability," and the views of Bichât were just as correct on this head, as any that have sprung from the excito-motory hypothesis. The language employed was bad, but the idea represented was not. We think that in this, as in several instances, the ardent advocates of the "excitomotory" doctrines have flattered themselves that they were doing more than they really were—have supposed that they were establishing new views when they were only using new words.

It does not appear to us that there are any further observations in this chapter which demand particular notice. We therefore pass to the succeeding one. It is entitled:—

B. Theory of the Functions of the Sympathetic Nerve.

It is, on the whole, the general opinion that the ganglia of the great sympathetic are the seat of some degree of independent power. Dr. Wilson Philip, indeed, and some other physiologists suppose that the ganglia derive their energy from the brain and spinal cord. Dr. Hall thinks that the ganglions of the great sympathetic, and of the cranio-spinal nerves, constitute that part of the nervous system which ministers immediately to the nutrition of the internal and external parts of the body.

The theory of the dependence of the ganglia of the sympathetic on the brain and spinal cord appears to be opposed by many analogical and by some positive considerations. The latter are, first, the non-existence of both the encephalon and spinal cord in some cases of monstrosity; and, secondly, some circumstances mentioned by the late Dr. Fletcher. Thus, we learn from him, that although the rudiments of the spinal cord and brain are among the first parts visible, yet, that the ganglions speedily acquire a greater degree of development, and are relatively larger in the fœtus than after birth, so that whilst the cerebro-spinal system is still obscure, the sympathetic and its ganglions are very distinct in the embryo. It is further remarked, that the first portion of this system which becomes obvious, is the cardiac ganglion. As regards those cases of monsters, in which it is said that no trace of a nervous system of any kind could be discovered, it is probable, as Dr. Fletcher remarks, that attention was particularly directed to the existence of the cerebro-spinal, rather than of the sympathetic system, and that the latter might in an imperfect state have been present, although not detected.

Mr. Grainger believes that the ganglia of the sympathetic present a structure which is, with one most important exception, identical with that of the spinal cord, when it is regarded in a collective form; that is to say, as consisting of an excito-motory and a cerebral portion. This structure is more or less obscured, by the dense neurilema which the ganglia possess, and by the cellular membrane which dips into their interior. But, Mr. Grainger observes that, we find in the ganglia:—

1. Grey matter.

2. Longitudinal and transverse commissural fibres, *ex. gr.* those joining the ganglions, in a longitudinal direction, forming the trunk of the nerve, as it is called; and those joining the ganglions, on the opposite sides of the body, as in the abdomen.

3. Fibres joining the sentient nerves, *ex. gr.*, those going to the nasal branch of the trifacial and posterior roots of the spinal nerves.

4. Fibres joining the motor nerves, as those going to the third and anterior roots of the spinal nerves.

5. Proper fibres.

Mr. Grainger remarks that:—

"The great distinction between the spinal cord and ganglions is, that, whilst the cerebral fibres of the former organ transmit impressions which excite sensation and the influence of the will, the cerebral fibres of the sympathetic, only convey impressions which excite pain, as in morbid states of the intestine, and the influence of the passions." 136.

This is true to a great extent, but we question whether it is so entirely. It is probable that the sympathetic system is the vehicle of agreeable as well as of painful sensations. The "comfort" experienced from satisfying the appetites, appears to be a species of pleasurable sensation, conveyed through the medium of the sympathetic system.

But we must proceed to Mr. Grainger's theory of the office of the ganglia of the sympathetic.

He conceives that they form a part, though in some degree an isolated one, of the excito-motory system. He adopts this opinion, because,—1, the quantity of grey matter contained in the ganglia seems to indicate that they are an independent seat of nervous energy; 2, the existence of the great sympathetic in all its integrity, in cases of deficiency of the cerebro-spinal axis, affords in itself a strong presumption that it forms a peculiar and independent division of the nervous system; 3, this view is further supported, by the very early appearance of the sympathetic ganglions in the embryo; and by their superior development, when contrasted with the cerebro-spinal system, in the fœtus and young child; or at those exact periods, in which the organs to which their nerves are distributed (those, namely, of nutrition), are so remarkable, for the activity of their functions; 4, as the actions of the excito-motory system are invariably the result of the application of a physical agent to the surfaces of the body, so "the contraction of all those organs to which the sympathetic is distributed, with the questionable exception of the kidneys, is incessantly being influenced by physical agents; that of the heart and blood-vessels, by the blood; of the intestinal canal, by the food; of the secreting canals, of the salivary glands, the pancreas, and the liver; also by the food, through the medium of their relations with the alimentary canal; of the testicle, in coitu; and of the uterus, by the fœtus."

Such are Mr. Grainger's reasons for concluding that the sympathetic is a part of the excito-motory system. Having gone so far, he endeavours, of course, to go farther. He thinks it necessary to discover the incident and reflex nerves by which its action is effected.

The sympathetic has, besides the commissural fibres and those by which it is connected with the cerebro-spinal system, a peculiar set of filaments. It is these which he conceives to constitute a true excito-motory system of nerves. If it was difficult to demonstrate the four orders of fibres in the spinal nerves, it must be impossible to distinguish the exact arrangement of the fibrils in the ganglia of the sympathetic. But fortunately there is a ganglion which suits Mr. Grainger's purpose admirably, and "seems to furnish" the clue he wants. It is the submaxillary ganglion. Its advantages result from the circumstance of its being, in a certain degree, isolated; and especially because the organ which receives the impression, the tongue, and those organs which contract, in consequence of that impression (the tube of the salivary glands), are separated from each other.

It will be necessary to extract Mr. Grainger's account of the anatomy of this ganglion, in order to lay his theory fully before our readers.

"The submaxillary ganglion," he says, "is neither placed on the chorda tympani, as Cloquet supposed; nor is it formed, as Cruveilhier asserts, by the most inferior fibrils of the lingual nerve. Professor Arnold, who has represented this nerve, has given the most accurate account of its connexions, which has yet been published; he has not, however, noticed all the branches of this little system, which may be received as the type of the great sympathetic. In reflecting upon this subject, I was induced to conclude, that, besides the branches which are furnished to the submaxillary gland, there must be others supplying also the sublingual gland; and, therefore, to determine this point, my colleague, Mr. Walker, dissected this ganglion, and the following is the result of a careful dissection. Many more branches than are figured by M. Arnold, are given off from the lower border of the ganglion, and enter the submaxillary gland; two or three others pass upwards, behind the gustatory nerve, then above and parallel to the Whartonian duct, into the substance of the sublingual gland; the latter twigs are not noticed by Arnold, Hildebrandt, Cruveilhier, Swan, nor, I believe, by any other anatomist. The branches, then, of the submaxillary ganglion, are,—1, to the great sympathetic; 2, to the chorda tympani; 3, from its upper and outer border twigs, to the gustatory nerve; 4, a second order of twigs, from the upper and inner edge to the gustatory nerve; 5, fibres to the submaxillary gland; 6, filaments to the sublingual gland. Notwithstanding the extraordinary number of different branches, each class of them is, doubtless, provided for a special purpose; and, without asserting any thing positively, it may be suggested that the fibrils of the first order are commissural branches, like those uniting the other ganglions together; that the second transmit the influence of mental emotions, which are capable of affecting all the organs supplied by the sympathetic, the effect, in this instance, being confined to causing a flow of saliva, on seeing or even thinking of food; that the third convey impressions to the brain, as in inflammation of the salivary glands, exciting pain; that the fourth are incident branches, arising from the surface of the tongue; that the fifth and sixth are reflex branches, going to the two glands. The flow of saliva, during mastication, is generally supposed to result from the motions of the jaw; or from a kind of continuous sympathy, caused by the mucous membrane of the mouth entering into these glands; but it is much more in accordance with the manner in which muscular contraction in general is determined, to attribute this phenomenon to the impression made on the nerves of the tongue, by the food exciting, through the medium of the ganglion, the contractility of the secreting canals; a supposition which is rendered the more probable, by the well-known fact that certain substances act as sialogogues, (tobacco, for example), by the irritation which they cause on the tongue." 141.

It must be admitted, that this anatomical explanation of the reflex function of the sympathetic appears, on a superficial examination, rather fanciful. But we leave it to its merits and its fate. Mr. Grainger observes, that, in the case of the saliva, there is an instance of a secretion flowing, in consequence of the contact of a physical agent, food, with what appear to be incident nerves. And on the whole he concludes with the majority of anatomists, that, in fœtal life, at all events, the processes of circulation, secretion, and nutrition are dependent on the agency of the sympathetic—an agency which he himself thinks must "almost certainly" be dependent on the "reflex principle." And, as a general law, he conceives that the involuntary organs of the circulation and secretion never contract but upon the application of a mechanical stimulus to the extremities of the nervous filaments. He winds up the chapter and the subject with the following remarks:—

"Many considerations, the principal of which have been stated, induce me to suppose:—

1. That the great sympathetic consists of several distinct nervous systems, each of which is endowed with an independent power.

2. That every ganglion is provided with incident and reflex fibres which are necessary to the exercise of its peculiar power.

3. That the contraction of all the organs which are in a more especial manner supplied by the great sympathetic, is in every instance excited through the agency of the incident and reflex nerves.

4. That the power of the ganglions is invariably excited by the application of a physical agent to the incident nerves, distributed to the internal surface of the heart, blood-vessels, intestines, and secreting canals of all glandular organs.

5. That the sympathetic ganglions are connected together by commissural fibres; and to the cerebrum by branches which join the sentient and motor cranio-spinal nerves, by which impressions are reciprocally transmitted between the brain and the organs supplied by the sympathetic." 143.

On a narrow inspection, this hypothesis does not contain much that was not previously suspected, nor prove much that was not proved before. A new language is employed, and incident and reflex chequer the several propositions. But, after all, the comparative independence of the ganglia—their indifferently understood connexion with the cerebro-spinal system—the influence of the contents of the viscera on their action through the medium of the impressions made by those contents on their surfaces—are *ideas* not rendered more familiar, not better established on more certain grounds, nor much more satisfactorily explained, than they were by Mr. Grainger's predecessors, or are by his contemporaries. We may be wrong, but we do not perceive much advance on our pre-existing knowledge in Mr. Grainger's views of the functions of the ganglia. The subject, however, is one of extreme intricacy, and, possibly, Mr. Grainger may have furnished the clue to its final disentanglement.

### C. THEORY OF MUSCULAR ACTION.

This forms the subject of the last chapter of the volume. Setting aside the prevailing opinions on the subject, opinions with which we need not trouble our readers, we may at once state Mr. Grainger's.

He believes that muscular contraction is only excited by three causes:—the first consisting of volition, the second of the reflex power possessed by the spinal cord and the ganglions of the great sympathetic; and the third by the passions, fear, joy, &c. The voluntary power is in Mr. Grainger's view the most limited, being confined to the muscles supplied by the cerebro-spinal axis, whilst the control of the reflex power and of the passions is exerted over every muscular organ in the body. In order to examine the matter more satisfactorily, Mr. Grainger notices separately the voluntary, the instinctive, and the involuntary motions.

A. *Voluntary Motion.* The gist of Mr. Grainger's observations on this head lies in the position that the actions of the voluntary muscles are not unfrequently excited, and due to the reflex function. The closure of the eye by the orbicularis on touching the eye-lash, is cited as a specimen. In other instances the motions are partly voluntary and partly excited, as in the familiar case of deglutition. Mr. Grainger contends that locomotion is in the same predicament, in other words, that walking depends in a great degree on the impression conveyed from the foot to the spinal marrow, and thence reflected to the various locomotive muscles. If this be the case there is no knowing where to stop, for all combined movements have as good or nearly as good a right to be excited as locomotion. As Mr. Grainger goes over ground which he and we have already trodden, we need say no more upon this head.

B. *Instinctive Motions.* On this Mr. G. does not deem it requisite to dwell.

C. *Involuntary Motions.* Two great theories agitate and have long agitated the physiological world on the cause of the involuntary motions. By the one theory, action of the involuntary muscles is supposed to depend on the immediate contact of certain substances (of the blood, for instance, with the heart); by the other theory, their action is attributed to impressions on the nerves by which they are supplied. Mr. Grainger's criticisms on the former, perhaps the most generally received, hypothesis, are so just that we transcribe them.

"The theory by which the contraction of the involuntary muscles is referred to immediate contact with their fibres, is, when carefully considered, very unsatisfactory. It may, in the first place, be observed, that in no organ does actual contact take place; there is always a membrane interposed between the fleshy fibres and the contents of the organ. But, even allowing that where the muscular planes, as in the intestine, are so thin as to allow the food to approach sufficiently near to make the requisite impression, how can this be supposed to happen in the left ventricle of the heart, where there is such great thickness of the parietes? The only manner in which it can be imagined that the fibres towards the outer surface, or pericardium, are capable of being stimulated, is by an impression transmitted through the cardiac nerves; but such an explanation belongs to another and different hypothesis. This subject may be elucidated by considering the action of two structures, both of which are employed in the same office, that of propelling the food along the alimentary canal. In the fauces, it is not supposed, that the numerous muscles employed in that action are made to contract by the actual contact of the food; indeed, the relative situation of the parts implicated, some of the muscles being placed so remotely in the neck, renders such contact physically impossible. It is, indeed, not known that the process of deglutition is excited; but then it is supposed that when the food descends rather lower, and reaches the pharynx, œsophagus,* and intestine, that a new principle of muscular action is called into operation, and that contraction is effected, not through the medium of the nerves, but by direct contact with the muscular substance. This idea has doubtless originated from the membranous form which the muscular structure assumes in the œsophagus and intestine, and partly from the difficulty of unravelling the nerves of the abdomen. It is not, however, probable, that parts so very analogous to each other, as the œsophagus and fauces, and which are supplied by the same nerves, should have their action excited in two different modes.

With respect to what is called sympathy, it may be observed in general, that

* Professor Müller has very justly questioned the correctness of Dr. Hall's original opinion that the action of the œsophagus is caused by immediate contact. Dr. Hall has since modified his views on this point.

it is a word to which the most vague meaning is attached, but which is nevertheless very commonly employed, as if it explained the cause of some of the most important phenomena of the economy, whilst it is nothing but a term by which those phenomena are designated. The continuous sympathy which is said to be the cause of the saliva flowing into the mouth when this cavity contains food, is but an expression by which is signified the effect produced in the salivary glands, by the impression made on certain nerves of the mucous membrane of the tongue. The same error which has prevailed on so many other subjects connected with the properties of the nervous system, is particularly exemplified in the present instance; for whilst it is believed that sympathy is but a modification of sensation, it has been proved that this form, or continuous sympathy, is displayed by the flow of the saliva, gastric juice, &c., when all sensation has been destroyed." 154.

It is almost needless to add that Mr. Grainger is one of those who attribute the contraction of the involuntary muscles to the ganglionic nerves, the operation being of that reflex character with the ganglia as central axes, which was described in the chapter on the functions of the sympathetic nerve.

One objection occurs to us. By Mr. Grainger's hypothesis the contraction of an involuntary muscle is of this nature. An incident fibre of the sympathetic nerve is excited by some stimulus—that incident fibre conveys the excitement to the ganglion—the ganglion thus excited originates the motive power—this is conveyed to the muscle by the reflex fibre—and so the muscle acts. Now it is clear that the ganglion in this view of the matter plays an essential part in the process, and if the ganglion be removed we do not see how the machinery of contraction is to work. If, in point of fact it *does* work, surely that must stagger our faith in the hypothesis.

This is not an imaginary difficulty. When the heart is removed from the body, and, of course, from connexion with the plexuses and ganglia, it still, if immediately immersed in warm water continues for a short time to contract with regularity. In Mr. Mayo's experiment, the auricular part of a heart removed from the body was separated from the ventricles, yet the alternate states of action and relaxation continued for a time to succeed each other. Now either the ganglia are not so necessary as Mr. Grainger supposes, or the nerves can retain the power communicated to them in some manner which Mr. Grainger has not included in his theory. That theory resembles in all essential respects, the theory of the reflex action of the spinal cord; the only difference being, that, in one case, the grey matter of the cord forms the centre, and, in the other, the sympathetic ganglia. Now, if in the former case, the communication with the cord is arrested, the reflex phenomena instantly cease—yet in the case of the organs supplied by the sympathetic, we do not find such to be the case. We mention these circumstances because they appear, at first sight, to be objections to Mr. Grainger's views.

This gentleman anticipates much from these views, and, perhaps, with reason. He has carried the reflex theory farther than most others, and when he proves, or endeavours to prove, that even locomotion is in a great measure a series of excited actions, it will be evident that he goes some length. And so in fact he does, for he believes it "quite possible," that "the arms, mouth, and stomach of the polypi may seize, swallow, and digest its prey without instinct, sensation, or volition."

We have now concluded the examination of this work. Its demerits being slight, we may dismiss them first. The main fault is frequent repetition. The same ideas, the same facts, often the same language, re-appear in chapter after chapter, and form a rather tiresome rechauffé. Perhaps the author leans too much upon the reflex theory, and considers it as a discovery of greater magnitude than it really possesses. But the merits of the book far outweigh such faults, and it must be considered not only a valuable addition to anatomical knowledge, but as valuable an one to physiology and philosophy. It will direct the minds of exact inquirers to the examination of the "excito-motory" doctrine, and especially to the anatomical basis upon which it rests. Whatever may become of the doctrine itself, an acquaintance with the latter must necessarily lead to the discovery of truth. Before we dismiss the subject for the present, we are tempted to subjoin the classification of the nervous system, embodied in the appendix to the work. It shews at a glance Mr. Grainger's views, and the reflex theory in its most recently developed state.

"CLASSIFICATION OF THE NERVOUS SYSTEM, ACCORDING TO ITS PHYSIOLOGY.

1. SOURCES OF POWER.

Grey matter of — A, Brain ; B, True Spinal Cord ; C, Ganglions.

II. CONDUCTORS.

A. Fibres of the Brain ; B, Fibres of the Spinal Cord ; C. Fibres of the Nerves.

III. DIVISIONS OF THE NERVOUS SYSTEM.

A. Cerebral System.

a. Convolutions of the Cerebrum and laminæ of the Cerebellum.
b. Sensiferous Nerves, comprising the true sensiferous fibres of—
1. Olfactory.
2. Optic.
3. Auditory.
4. Gustatory.
5. Nerves of the Skin, viz. Cerebral Fibres of the portio major, of trifacial, and of the posterior roots of the Spinal Nerves.
6. Nerves of the Mucous Membrane, viz. Cerebral Fibres of the portio-major, of trifacial, glosso-pharyngeal, pneumo-gastric.
7. Sentient Fibres of the great Sympathetic, supplying the organs of organic life.
c. Volition Nerves, consisting of the cerebral fibres of
1. Oculo-motor.
2. Pathetic ?
3. Portio Minor of Trifacial.
4. Abductor.
5. Portio dura.
6. Glosso-pharyngeal.
7. Pneumo-gastric.
8. Accessory ?
9. Sublingual.
10. Anterior roots of the Spinal.

- B. Excito-motory System.
  - 1. True Spinal System.
    - a. Grey matter of the true Spinal Cord.
    - b. Incident Fibres of
      - 1. Optic.
      - 2. Auditory.
      - 3. Portio major of fifth.
      - 4. Glosso-pharyngeal.
      - 5. Pneumo-gastric.
      - 6. The posterior roots of the Spinal.
    - c. Reflex Fibres of
      - 1. Oculo-motor.
      - 2. Pathetic?
      - 3. Portio Minor of Trifacial.
      - 4. Abductor.
      - 5. Portio dura.
      - 6. Glosso-pharyngeal.
      - 7. Pneumo-gastric.
      - 8. Accessory.
      - 9. Sublingual.
      - 10. Anterior roots of the Spinal.
  - 2. Sympathetic System.
    - a. Grey substance of the Sympathetic Ganglions.
    - b. Incident branches of
      - 1. The Ganglions of the trunk of the great Sympathetic.
      - 2. Cavernous?
      - 3. Lenticular?
      - 4. Spheno-palatine.
      - 5. Optic?
      - 6. Submaxillary.
      - 7. Cardiac.
      - 8. Semilunar.
    - c. Reflex branches of the same Ganglions." 159.

### II. On the Ganglionic Enlargement of the Pneumo-Gastric Nerve.

The paper on this subject, which is brief, is contained in the Number of the Guy's Hospital Reports for October of last year. The author, Mr. Edward Cock, professes that he acts principally as the amanuensis of Sir Astley Cooper, following out the zealous Baronet's views, and enlarging somewhat his exclusive discovery. That discovery and those views, as well as Sir Astley's property in them, may be best introduced to our readers by a copy of the note in which they were introduced to Mr. Cock.

"MY DEAR EDWARD, "*April* 18, 1837.

"I have sent you the *superior laryngeal ganglion* of a rabbit, which I found last year (February 1836), whilst making the experiments I have published in the Guy's Hospital Reports on the Compression of the Carotid and Vertebral Arteries. I always thought it an objection to my friend Sir Charles Bell's opinion of the ganglia giving sensibility, that the laryngeal nerve, going as it does to parts of the most sensitive description, was not ganglionic. It gave me much pleasure to find this ganglion. If you put the nerve in water for five minutes, you will see the usual colour of a ganglion in the enlarged part from which the laryngeal nerve springs.

"Yours affectionately,

"ASTLEY COOPER."

It is well known that the pneumo-gastric nerve presents a slight enlargement at the external basis of the skull, just as it is leaving the foramen lacerum. This has been called a ganglionic enlargement; but no great degree of anatomical or of physiological importance has been given to it. The general opinion with respect to the functions of the pneumo-gastric nerve assign to it the office of regulating, more or less, respiration, deglutition and digestion, of communicating its peculiar sensibility to the mucous membrane of the larynx, and of controlling the action of the muscles of that part. The sensibility of the larynx, and the action of its muscles, are believed to depend on the superior and inferior laryngeal branches of the pneumo-gastric, though the precise part that is played by each is far from undisputed. Mr. Cock, indeed, assures us that:—

"The distribution of the laryngeal nerves is, I believe, incorrectly given in all anatomical works. My colleague, Mr. Hilton, has lately taken much pains in the investigation of this subject; and the result of his dissections shew that the superior laryngeal nerve (after it has pierced the thyro-hyoideal ligament) gives off no muscular filaments whatever, but is entirely distributed to the mucous membrane. The crico-thyroideus is, therefore, the only laryngeal muscle supplied by it in the human subject; and in some animals, the crico-thyroidean twig will be found to arise, not from the superior laryngeal, but from the trunk of the pneumo-gastric itself. All the proper muscles of the larynx, with the exception of that just mentioned, receive their nerves from the recurrent branch alone." 313.

Thus, if these dissections be accurate, there can be little question that the superior laryngeal nerve is one of sensation, and the inferior one of motion. If we now examine, says Mr. Cock, the spot where the nervus vagus leaves the surface of the medulla oblongata, we shall find that the nervous fibrillæ which compose it come off in close apposition; on the one hand, with the corpora restiformia, or common sensory columns of the spinal cord; and on the other, with those fibres of the corpora pyramidalia, or anterior motor columns, which Mr. Solly has traced, and described as passing into the cerebellum: so that, besides the origin which the pneumo-gastric is supposed to derive from the olivary bodies, comprising, no doubt, the greater portion of the nerve, and constituting its specific character, it likewise possesses every facility of position for deriving fibres from the anterior and posterior columns, or the motor and sensory tracks of the medulla spinalis. In short, Mr. Cock considers the superior laryngeal branch of the pneumo-gastric as a nerve of common sensation, and derived, with the exception of a few motor filaments, from the sensory column of the spinal cord—that it is analogous to the posterior roots of the spinal nerves—and that the ganglion is the ganglion belonging and appended to the sensory portion of the nervus vagus. In a note, Mr. Cock lays before the reader the following results of the various dissections he has made:—

"From the dissections I have made, I have every reason to believe that the ganglion does not belong exclusively to the laryngeal branch, but extends its influence to numerous other filaments included in the trunk of the par vagum, and affording to the lungs, to the pharynx, œsophagus, and stomach, that faint but peculiar sensibility which they appear to possess—filaments which impart to the stomach the sensation of fulness when that organ has been distended with food; to the lungs, the 'besoin de respirer,' and the sensory functions necessary for respiration alluded to by Bichat. In the sheep, where I was enabled to unravel the fibres of which the par vagum is composed, and trace with considerable accuracy their course and their connexion with the ganglion, I found the following arrangements to exist:—The pneumo-gastric trunk, as it left the base of the scull, might be said to consist of two orders or sets of filaments; viz., the ganglionic, and the ganglionless. The former terminated in the ganglion, where their fibrous character became lost after the manner of the posterior roots of the spinal nerves: the latter were continued downwards beyond the ganglion, having merely a cellular connexion with it, and resembled in this respect the motor portion of the fifth pair. Lastly, from the ganglion arose two sets of nerves: the one constituted the laryngeal; the other joined the ganglionless filaments mentioned above, and formed part of the trunk of the par vagum, descending to the chest. A careful dissection will bring to light a similar arrangement in the human subject, the horse, the ass, and probably in other animals. (*Vide* Diagrams 5, 6, 7.) I may also observe, that the laryngeal nerve appeared to derive some very minute fibrillæ from the pneumo-gastric trunk above the ganglion. These might be either specific respiratory filaments, or motor-muscular filaments, perhaps both." 315.

Mr. Cock remarks, in continuation, *that* the ganglion seems proved to belong to that class of ganglia which, apparently, are necessary appendages to all nerves of common sensation, because, both in colour and texture, it resembles those placed on the posterior roots of the spinal nerves: and *that* it belongs more particularly to the superior laryngeal nerve, because, in the larger animals greater accuracy of examination is practicable, the laryngeal branch may be seen to come off distinctly from the ganglion, and the position of the latter varies according to the origin of the nerve. Thus in the human subject, the ganglion is situated immediately at the base of the scull, and it is there that the laryngeal nerve is sent off. In the rabbit, the ganglion, and consequently the point where the laryngeal branch is detached from the pneumo-gastric trunk, will be found much lower down, or nearly in a line with the upper edge of the thyroid cartilage. In the dog, the ganglion is placed close to the lacerated opening, whence the laryngeal nerve descends very obliquely: in the ass, the latter is given off below the level of the larynx, and ascends to pierce the thyroid cartilage.

"The shape of the ganglion presents great varieties in different animals. In the dog, cat, rabbit, and rat, it is rounded and bulbous, projects considerably from the pneumo-gastric trunk, and is immediately recognised on laying bare the nerve.

In the human subject, in the horse, the ass, and the sheep, it is more or less elongated or spindle-shaped; and is in great measure concealed by nervous fibrillæ, which pass over its surface without being connected to it, and which must be turned to one side, before the body can be distinctly brought into view. Generally speaking, the length of the ganglion will be found to bear a certain proportion to the length of the neck of the animal; and the varieties of shape and position which it assumes in different animals has probably no other object than to adapt it more conveniently to the surrounding parts." 316.

The paper is accompanied with a few diagrams, representing the ganglion in the rabbit, the ferret, the guinea-pig, the dog, the ass, the sheep, and the human subject.

As far as Mr. Cock has been enabled to ascertain, the glosso-pharyngeal nerve appears to be furnished with a ganglion, similar in all respects to that upon the nervus vagus.

The preceding paper is not an uninteresting contribution to our knowledge of the anatomy of the nerves.

### III. On the Distribution and Probable Function of the Superior and Recurrent Laryngeal Nerves. By Mr. John Hilton.

Mr. Hilton confines himself to those portions of the superior and inferior laryngeal nerves, which relate more particularly to the functions of the larynx. He does not therefore allude to the former nerve until it gives off the crico-thyroid branch, nor to the recurrent nerve until it arrives at the middle of the trachea.

"The superior laryngeal nerve, after detaching from its fibrous sheath the crico-thyroideal* branch of the pneumogastric traverses the fibrous tissue occupying the interval between the thyro-hyoideal round, and the thyro-hyoideal broad ligaments: here some filaments are thrown into the cellular and fatty tissue investing the nerves and surrounding parts; after which, the distribution may be systematized into, 1. Ascending; 2. Transverse; and, 3. Descending.

1st. The ascending branches are from eight to ten in number: some of them take their direction upwards, forwards, and outwards, to the lateral fræna of the epiglottis, and the tissue immediately below the tonsil gland, where they are lost. Others pursue their course upwards and inwards, towards the epiglottis: they first pass rather on the glossal aspect of its margin, where some of them terminate; whilst the majority either perforate the epiglottis, or cross its margin in deep fissures, and are then very minutely distributed to the mucous membrane, submucous cellular tissue, and glands covering its laryngeal aspect.

The 2d, or the transverse filaments, enter the aryteno-epiglottidean folds; where two of them follow the transverse direction, and supply the cellular tissue at the anterior part of the root of the epiglottis; and then pierce it, to supply the laryngeal aspect of the same part of the epiglottis. Two or three small filaments of this transverse division of the nerve ramify amongst the glands; and appear to supply them, at the summit of the external wall of the pouch, which I discovered, and have described in this Number of our Hospital Reports. Other filaments of the same transverse series cross over the upper part of this sac; then descend, inclining forwards and inwards, to the anterior part of the rima-glottidis: some of them supply the anterior and inner aspect of the sac, the glands upon it, and the mucous membrane on the superior chorda vocalis: the others, having arrived at the anterior angle of the rima-glottidis, near the apex of the epiglottis, communicate, in the median line, with the corresponding filaments from the opposite nerve; and some fibrils can be traced to the anterior extremity of the inferior chorda vocalis.

The 3d, or descending set, or rather branch (for the filaments comprising this series continue congregated into one chord for more than half an inch, and then diverge), is contained in the posterior part of the aryteno-epiglottidean fold, following its direction to the outer side of the arytenoid cartilage; when one filament continues vertical between the mucous membrane and crico-arytenoideus, and communicates with the posterior branch of the recurrent nerve. Several filaments are lost in the submucous tissue covering the arytenoid cartilages, and also that, between them both, on the laryngeal and pharyngeal surfaces; the latter, as far the lower edge of the cricoid cartilage. There are two filaments which wind round the external edge of the arytenoid cartilage, and enter the upper part of the arytenoideus-transversus muscle. one of these, the smaller, joins the recurrent filament to this muscle; the other curves round the posterior and internal edge of the arytenoid cartilage, and then, descending along the inner side of its base, obliquely downwards and forwards, is distributed, by minute fibrillæ, upon the inferior chorda vocalis and the membrane lining the internal surface of the cricoid cartilage." 516.

The Inferior or Recurrent Nerve, after taking a course, and sending branches to the œsophagus, &c. that we need not stop to describe, finally perforates the inferior constrictor of the pharynx; then rests, covered by mucous membrane, upon the posterior part of the crico-thyroideal articulation, in a groove between the inferior cornu of the thyroid cartilage and the crico-arytenoideus posticus, to which muscle it sends four or five filaments: one of them passes obliquely upwards between this muscle and the cricoid cartilage; crosses the upper edge of the cartilage; then enters the arytenoideus transversus, supplies it and the arytenoidei obliqui, and joins in the transverse muscle a corresponding branch from the recurrent of the opposite side, and a branch of the superior laryngeal.

"The further distribution of the recurrent nerve is by separate filaments, which enter, on their external aspects, the crico-arytenoideus lateralis and thyro-arytenoideus muscles. 'Two of the filaments which pass into the last-mentioned muscle go through it to its upper edge, and supply the aryteno-epiglottideas, superior and inferior." 518.

"In conclusion," says Mr. Hilton, "I think we may abstract from the preceding facts two highly interesting and extremely important inferences: 1st, That the superior laryngeal nerve is a nerve of sensation; because, independent of the crico-thyroideal nerve—for an explanation of which I must refer to Mr. Cock's Paper on this subject—it is distributed exclusively to the mucous membrane, cellular tissue and glands. 2dly, That the inferior or recurrent nerve must be the proper motor nerve to the larynx; as it alone supplies all the muscles which act immediately upon the column of air passing to and from the lungs." 518.

### IV. Description of the Sacculus or Pouch in the Human Larynx. By Mr. John Hilton.

Mr. Hilton appears to consider this pouch no trifling matter, for he starts by saying, "I must denominate" it "*my* laryngeal pouch;" and he speedily takes occasion to assure us that he intends "very shortly to bring forward the *whole* subject in a more extended form, embracing the physiology." We are probably on the eve of a great work. At the end of the paper our expectations are a little damped, by the announcement, on our author's part, that his discovery was not quite what he had anticipated, the "pouch" having been described, though not very completely, by Galen, Morgagni, and M. Savart. We don't see how, after this, the pouch can be called Hilton's pouch; but it might, not inconveniently, be denominated "the pouch of Messrs. Galen, Morgagni, Savart, and Hilton." Thus each gentleman would get his share of immortality.

Mr. Hilton would suggest for *general adoption* the nomenclature which he employs himself; a reasonable request, which many authors make, and some are disappointed in obtaining. Seriously, we would advise Mr. Hilton to adopt a more subdued tone in the publication of his anatomical discoveries. Had he made out the great facts which have been promulgated by Bell or Magendie, he could hardly have adopted a loftier tone. As it is, it is rather too much in King Cambyses' vein. We will present Mr. Hilton's account of the pouch, as the modern works upon anatomy contain no accurate notice of it, though Cruveilhier briefly describes it.

Mr. Hilton employs the following terms in the following sense :—

"The superior opening of the larynx I shall call aryteno-epiglottidean, from its position; the inferior aperture between the inferior chordæ vocales, the rima ventriculi laryngis; the large or general cavity of the larynx, into which these open, ventriculus laryngis; the depression between the superior and inferior chordæ vocales, on each side, the fossa elliptica ventriculi; and the pouch I shall describe as the sacculus laryngis, or true laryngeal pouch, which terminates below, upon the fossa elliptica.

There are also two other depressions in the ventricle of the larynx, one on each side of the epiglottis, between its edge and the superior chorda vocalis; these depressions I term the fossæ superficiales ventriculi. Into these fossæ superficiales, the submucous glands, arranged on each side, at the edge of the epiglottis, pour their secretion." 520.

We now pass to the description of the laryngeal pouch. Mr. Hilton describes it as extending upwards, on each side, from the fossa elliptica or space between the superior and inferior chordæ vocales, interposed between the internal surface of the ala of the thyroid cartilage and the ventricle of the larynx, terminating below upon the fossa elliptica, and bounded above by a large quantity of fat; and its superior part is crossed from behind, forwards, by the aryteno-epiglottidean folds. The pouch averages about half an inch, or more, in height; and if distended, reaches the upper edge of the thyroid cartilage. Its shape is not always the same: sometimes it is nearly conical, with its base placed inferiorly; sometimes pear-shaped, with its broader part superiorly; occasionally nearly cylindrical, and generally curved upon itself slightly backwards.

Mr. Hilton goes on to state that the opening into the fossa elliptica is nearly oval when the chordæ vocales are stretched backwards, but more circular if they are relaxed;—that the opening of the pouch is provided with two small semilunar folds of membrane, placed anteriorly and posteriorly with respect to the centre of the aperture;—that an extension of the pulmonary mucous membrane entirely lines the pouch, which is perforated by very numerous and minute openings, the terminations to the excretory tubes from the glands which surround and belong to this pouch, pouring their secretion into it;—that nearly the whole of the exterior of the pouch is surrounded by a peculiar fat, which conceals from view its proper glands.

Below the aryteno-epiglottideus muscle, as it is usually described, there is another muscle, to the sole property in which Mr. Hilton lays claim, and which he terms the *aryteno-epiglottideus inferior*.

"The *aryteno-epiglottideus inferior* is easily brought into view, by taking off the mucous membrane of the ventricle of the larynx, immediately above the superior chorda vocalis, with a few small mucous glands which open upon it, some cellular tissue, and a few filaments of the superior laryngeal nerve: the muscle will then be seen passing from the arytenoid cartilage to the lower part of the epiglottis.

The muscle arises, by a narrow and fibrous origin, from the arytenoid cartilage, just above the arytenoid attachment of the superior chorda vocalis: it passes forwards and a little upwards, and, becoming expanded, covers the superior half, or sometimes two superior thirds, of the pouch, on its laryngeal surface; and is inserted, by a broad attachment, into the edge of the epiglottis. The nerve supplying this muscle enters its upper and outer edge: it is derived from the branch of the motor, or recurrent laryngeal nerve, which supplies the thyro-arytenoideus. Its functions appear to be, to compress the subjacent glands, which open into the pouch; to diminish the capacity of that cavity, and change its form; to approximate the epiglottis and the arytenoid cartilage; and it will also have the effect of raising the surface of the fossa superficialis."

521.

On removing the aryteno-epiglottideus inferior, we come to a fibrous membrane investing the pouch and its glands, and forming an internal and superior support to the former.

On the external, or thyroideal, aspect of the pouch, is another muscle—the *thyro-arytenoideus,* which, from its situation and attachments, is calculated to act directly upon the pouch.

"The glands belonging to, and proper to, the laryngeal pouch* are very numerous; as many as sixty or seventy may be distinguished. I have succeeded in injecting these glands with mercury. It is easily accomplished, in a fresh larynx, by submersing it in water for a few hours; then pressing the water out of the pouch; after which, the pouch is to be filled with mercury, when well-adjusted pressure with the fingers will be sufficient to inject nearly all the glands.

These glands are not all of the same size or form: some are made up of several small lobes; the duct from each lobe terminating in a common excretory tube, which perforates the parietes of the pouch. The larger of these glands are situated at the outer, upper, and anterior surfaces of the pouch; the inner or laryngeal aspect being occupied, generally, by small glands, each having a distinct excretory tube emptying itself into that part of the pouch nearest to it."

522.

The *nerves* of the pouch and of its glands are derived from the superior laryngeal.

Mr. Hilton concludes that the offices of such a pouch must be very important. They may, however, be easily summed up—to lubricate the chordæ vocales. How the arrangement of the pouch itself, of its muscular fibres, of its glands, of its fibrous membrane, and of its valvular sort of orifice, contributes to this end, we refer the curious to Mr. Hilton's paper for information.

V. THE CYCLOPÆDIA OF ANATOMY AND PHYSIOLOGY. Edited by ROBERT B. TODD, M.D. &c. &c. Part XIII. Illustrated with numerous Engravings. February 1838.

WE are glad to perceive that this excellent work is continued. Unforeseen circumstances must have given it a shock, from which the energy of its editor was requisite to revive it. Let us hope that it has been revived

* "By employing the term 'glands proper to the pouch,' it will be understood, that I do not include or refer to the glands, hitherto described by anatomists, termed epiglottidean or arytenoidean glands."

effectually. The present number is not deficient in the signs of vitality and animation; on the contrary, it is redolent of life. This Cyclopædia has become almost a national work. It would be disgraceful to the profession in this country where it not patronised. We have no doubt that the uncertainty which hangs over the continuous publication, and the definite termination of works brought out in parts, has tended to damp the ardour of purchasers. When the work is complete we hope and we believe its sale will be proportioned to its merits, which are great.

The principal articles in the present part are—Gasteropoda, by Mr. T. Rymer Jones—Organs of Generation, by the same gentleman—and Physiology of Generation, by Dr. Allen Thomson. We shall present some notice of the latter article, which is best adapted for our pages. Our object will be to offer a sketch of the principal facts connected with the subject, which are now admitted by anatomists and physiologists.

### Physiology of Generation.

There is no single formula which will express the mode of generation in all the classes of animals—there are no circumstances in which all agree.

"Some animals, for example, are propagated by the division of their whole bodies into pieces, each of which by a peculiar change becomes an independent individual entering upon a new life. Others arise like the parts of a tree by buds which remain for a time attached to the parent stem, and being afterwards separated from it assume an independent existence. A third class of animals have the power of forming and throwing off from their bodies a small portion of organized matter, which, though at the time of its separation from the parent, not resembling it either in form or organization, is yet possessed of the power of living for itself, and, after passing through a variety of successive changes of growth and evolution, of at last acquiring the exact semblance of the parent by which it was produced. In a fourth and last class, comprehending much the greatest number of animals, the function of reproduction involves a greater complication of vital processes than in the three other classes above alluded to. The union of two individuals of different sex becomes necessary, and the young owe their origin to the evolution of a more complex organized structure termed the egg, which is formed in and separated from the body of the female parent, and is the product of the union of the male and female of all animals in which the distinction of sex exists. The ovum or egg is most familiarly known to us in the eggs of domestic birds, to which the product of sexual union in all animals belonging to this fourth class bears a strict analogy in every essential particular." 425.

Though the complication of the generative process is usually proportionate to that of the organization of the animal, this is by no means universally true. Yet the function of reproduction will always be found to exert so important an influence on the economy, as to constitute one of the fundamental divisions in a classification of the vital processes.

The act of generation is highly curious, and no less curious than obscure. The inquisitiveness of science, and the passions of the vulgar, have alike made this a field for research, for wonder, and for fancy. The speculations of philosophers have been greedily swallowed by the ignorant and the credulous mob, and a mass of absurdity, or at all events of the most crude hypotheses has received extensive currency and credence. The day has arrived for examining this, as well as the other phenomena of life, and if, in this as in other things, we soon discover the point at which our researches are arrested by the barrier of "ultimate facts," we discover also that generation is scarcely more mysterious than nutrition, nor more wonderful than the other phenomena of life, which are all wonderful.

Generation cannot be properly understood, if examined only in a solitary class. The mind, from contemplating an extensive object in a single point of view, receives a partial, perhaps, an erroneous idea, and is apt to generalize on an imperfect premiss. This has been eminently the case in this instance. The vast number of the theories of generation, precludes even their recital. Drelincourt, an author of the last century, says Dr. Thomson, brought together so many as two hundred and sixty-two "groundless hypotheses," concerning generation from the writings of his predecessors, "and nothing is more certain," quaintly remarks Blumenbach, "than that Drelincourt's own theory formed the two hundred and sixty-third."

1. *Classification of the Theories of Generation.*—Of these theories, writes Dr. Thomson, two principal classes may be distinguished, according as they more directly relate, 1st, to the action of the parent organs, or 2d, to the changes in the egg belonging to the formation of the new animal. Of the first of these classes of theories Haller made three divisions, according as the offspring is supposed to proceed, 1st, exclusively from the organs of the male parent, 2d, entirely from those of the female, or 3d, from the union of the male and female products. The second class of these theories, that, viz. which relates more particularly to the formation of the new animal, may be arranged under two heads, according as the new animal is supposed, 1st, to be newly formed from amorphous materials at the time when it makes its appearance in the egg, or 2d, to have its parts rendered visible, by their being expanded, unfolded, or envolved from a previously existing though invisible condition, in the germ.

"The greater number," continues our author, "of the older theories of generation may then be brought under one or other of the above-mentioned divisions, viz. the theory of the Ovists, of the Spermatists, that of Combination, Evolution, or Epigenesis.

According to the first-mentioned of these hypotheses, or that of the Ovists, the female parent is held to afford all the materials necessary for the formation of the offspring, the male doing no more than awakening the formative powers possessed by, and lying dormant in, the female product. This was the theory of Pythagoras, adopted in a modified form by Aristotle; and we shall afterwards see that it resembles most closely the prevailing opinion of more modern times. The terms, however, in which some of the older authors expressed this theory are very vague, as, for example, in the notion that the embryo or new product 'is formed from the menstrual blood of the female, assisted by a sort of moisture descending from the brain during sexual union.'

According to the second theory, or that of the Spermatists, among the early supporters of which Galen may be reckoned, it was supposed that the male semen alone, furnished all the vital parts of the new animal, the female organs merely affording the offspring a fit place and suitable materials for its nourishment. Immediately upon the discovery of the seminal animalcules, these minute moving particles were regarded by some as the rudiments of the new animal. They were said to be miniature representations of men, and were styled homunculi, one author going so far as to delineate in the seminal animalcule the body, limbs, features, and all the parts of the grown human body. The microscopic animalcules were held by others to be of different sexes, to copulate, and thus to engender male and female offspring; and the celebrated Leeuwenhoek, who was among the first to observe these animalcules, described minutely the manner in which they gained the interior of the egg, and held, that after their entrance they were retained there by a valvular apparatus.

The theory of the Syngenesis or Combination seems to have been applied principally to the explanation of reproduction of quadrupeds and man, the existence and nature of the ova of which were involved in doubt. This hypothesis consists in the supposition that male and female parents both furnish simultaneously some semen or product; that these products, after sexual union, combine with one another in the uterus, and thus give rise to the egg or structure from which the fœtus is formed. In connexion with this theory we may also mention that of Metamorphosis, according to which a formative substance is held to exist, but is allowed to change its form in order to be converted into the new being; as also the notion of Buffon that organic molecules universally pervade plants and animals, that these are all endowed with productive powers, that a certain number are employed in the construction of the textures of organized bodies, and that in the process of generation the superabundant quantity of them proceeds to the sexual organs and there constitutes the rudiments of the offspring." 427.

The theories of generation, prior to the time of Harvey being based on few facts were either vague, or actually erroneous. When the Harveian dogma—"omne vivum ex ovo"—was generally admitted as established, theories changed objects, and the development of the fœtus in the egg, and afterwards, became the subject of inquiry and of speculation. Then arose the discussion on the respective merits of Epigenesis and Evolution, a discussion which can scarcely be considered as completely set at rest.

About the middle of the last century, Caspar Wolff supported the doctrine of Epigenesis in an elaborate and philosophical manner. He held that no appearance of the new animal is to be found in the perfect impregnated egg before the commencement of incubation, but that when the formative process is established by the influence of heat, air, and other circumstances necessary to induce it, the parts of the fœtus are gradually put together or built up by the apposition of their constituent molecules. Haller referred both to his own observations on the chick and to a variety of collateral arguments in support of the system of Evolution, holding that when the fœtus makes its appearance in the egg, it does so merely in consequence of the enlargement or evolution of its parts, which pre-exist, though in an invisible condition, in the egg. Bonnet carried this theory farther than any one else, but trusting mainly to the observations of Haller on the formation of the fœtus, he supported his overdrawn views on highly hypothetical reasoning. Bonnet, in what is termed the theory of Emboitement, held not only that the whole of the parts of the fœtus pre-exist in the egg before the time of their appearance, but also that the germs of all the animals which have been or are to be born, pre-exist from the beginning in the ovaries of the female; that the genital organs of the first parents of any species, therefore, contain the *germs* of all their posterity; that these germs lie dormant in their abode until one or more are aroused by the exciting influence of the male; and that consequently there is not in nature the new formation of any animal.

This is "going the whole hog." The nature of the inquiry renders a determinate conclusion difficult, if not impossible. Modern investigations have thrown the preponderance of probability on the side of Epigenesis. But, for practical purposes, it is better to disregard all theory, and, studying the phenomena of the progress of the embryo to maturity, to apply to them the term Development, which expresses that progress unfettered by the weight of speculation. The following sentiments of Harvey are remarkable for their quaint and noble simplicity. Dr. Thomson quotes them from Harvey's 54th Exercitation.

"But as in the greater world we say *Jovis omnia plena*, all things are full of the Deity, so also in the little edifice of a chicken, and all its actions and operations, *digitus Dei*, the finger of God or the God of nature doth reveale himself." "A more sublime and diviner artificer (than Man is) seems to make and preserve man; and a nobler agent than a cock doth produce a chicken out of the egge. For we acknowledge our omnipotent God and most high Creator to be every where present in the structure of all creatures living, and to point himself out by his workes; whose instruments the cock and hen are in the generation of the chicken. For it is most apparent, that in the generation of the chicken out of the egge, all things are set up and formed, with a most singular providence, divine wisdom, and an admirable and incomprehensible artifice." 429.

2. *Modes of Generation.* Whatever the precise mode of reproduction may be, the general law of Nature is, that organized beings proceed from organized beings, the two standing to each other in the relation of parent and progeny. But this general law is supposed not to be free from exceptions, and some of the simpler animals and plants are conceived to be produced spontaneously from decaying organic substances, under particular and appropriate circumstances. This species of production has received the name of spontaneous generation.

*a. Spontaneous Generation.* This is imagined to occur among cryptogamic plants of the nature of mould, small microscopic animalcules formed in infusions of decaying organic matters, and the entozoa which live in the bodies of other animals. The inquiry into the reality and the nature of this process is beset with many and almost insuperable difficulties. We have not space at present to go into the reasoning and experiments which have been advanced on the opposite sides of this question, and we shall merely observe that, on the whole, the evidence, so far as it goes, and imperfect as it is, inclines to the side of the occasional occurrence of spontaneous generation in the simplest forms of organic life. But this is the exception, not the rule, and, once formed, these animals or vegetables propagate their species in the ordinary manner. Having made this observation, we shall content ourselves with quoting the brief considerations urged by Dr. Thomson in favour of the occurrence of spontaneous generation in infusoria, mould, &c.

"Firstly, those organic matters which are most soluble in water, and at the same time most prone to decomposition, give rise to the greatest quantity of animalcules or cryptogamic plants.

Secondly, the nature of the animalcule or vegetable production bears a constant relation to the state of the infusion, so that, in similar circumstances, the same are always produced without this being influenced by the atmosphere. There seems also to be a certain progressive advance in the productive powers of the infusion, for at the first the animalcules are only of the smallest kinds or Monades, and afterwards they become gradually larger and more complicated in their structure; after a time the production ceases, although the materials are by no means exhausted. When the quantity of water is very small and the organic matter abundant, the production is usually of a vegetable nature; when there is much water, animalcules are more frequently produced.

Thirdly, on the supposition that infusory animalcules are developed from ova, it is necessary to conclude, from the experiments already referred to, that these ova are in some instances derived from the atmosphere, but yet the number of Infusoria is by no means in direct proportion with the quantity of air. We are also reduced to the necessity of holding that every portion of the atmospheric air is equally impregnated with infusorial germs or ova, and that these bodies may remain for years dissolved, as it were, or invisibly suspended in the atmosphere, and in a perfectly dry state—a supposition contrary to analogy, and not fully warranted by the fact that Vibriones may be resuscitated by means of moisture after they have been kept in a dry state for long periods.

Fourthly, it may be remarked that the existence of ova, as belonging to many of the Infusoria, is entirely hypothetical, since most of these animals are known, when once formed, to propagate by other means, as by the division of their whole bodies or by budding.

The production of infusorial animalcules from solutions of granite, silex, &c. recently described by Mr. Crosse, we have no hesitation in pronouncing to be either a mistake, or the result of changes occurring in admixed particles of organic matter." 431.

The Entozoa are still more decisive proofs of spontaneous generation.

b. *Unequivocal Generation* may be either non-sexual, or sexual. Non-sexual generation occurs among the simplest animals only—sexual, among the higher class of invertebrated and among all the vertebrated animals.

*Non-sexual Generation* may take place either by *division;* or, by *attached buds;* or, by *separated gemmæ.*

c. *Fissiparous Generation.* The most common form of generation by division, or fissiparous generation, is met with in some of the simpler infusoria, but occasionally occurs in animals higher in the scale. It consists in the division of the parent animal body into a certain number of subordinate masses, each of which, being endowed with independent life, becomes a new individual similar to that of which it originally formed a part. In some of the Infusoria in which the process of subdivision has been minutely observed, fissures are seen to form in the sides of the animal which is about to be reproduced; these fissures gradually enlarge, and meeting with one another, completely separate the parts. In one kind of fissiparous generation the parent body is split into irregularly shaped masses, in some two in number, in different others, four, six, eight, or twelve, and in one, the Gonium pectorale, into as many as sixteen. Each of the subordinate masses, when first separated from its fellow, has an irregular shape, from which it gradually passes into the form and size of its parent.

In a second form of the fissiparous generation, the infusorial animal is divided into two equal and symmetrical halves; in some instances in a longitudinal direction, as in Baccillaria and some Vorticellæ; in others in a transverse direction, as in Paramœcium, Cyclidium, and Trichoda.

"A fissiparous kind of generation is not, however," continues Dr. Thomson "confined to the Infusoria, but occurs also in some of the Cestoidea and Annelida. The most remarkable example is met with in the Nais and Nereis. In the first of these genera, a small portion separated from the tail becomes the new animal. Before the actual separation of this caudal portion, it is marked off from the rest by a notch, and there are gradually formed on its sides the joints, hairs, and other indications of the organs of the complete animal in miniature. The notch enlarges, and the part at last drops off capable of independent existence. In the Nais, that part of the offspring by which it is attached to the parent becomes the head, and in this way, according to the singular notion of Gruithuisen, who observed this sort of reproduction with attention, the tail of a Nais may be considered as gifted with perpetual life, since this part is extended into each of the new descendants." 432.

The multiplication of individuals by division, which happens occasionally, or by accident, in several of the lower animals, may be regarded as analogous to fissiparous generation. The most remarkable instances are in Polypi, Entozoa, and Annelida. When the Hydra viridis is cut through either longitudinally or transversely, each segment continues to live and grow, and is gradually furnished with those parts of the body of which it was deprived by the division.

*d. Gemmiparous Generation.* In this the second form of non-sexual reproduction, the new individual grows upon the parent as a bud or sprout, at first exhibiting little appearance of the form or structure of the perfect animal; gradually assuming its form while still attached to the parent stem; and being afterwards separated to enjoy independent existence.

"The best known examples of this kind of generation occur in the polypine and coralline animals, and the process has been observed with great attention by Trembley in the Hydra viridis. In this animal the young polype makes its first appearance as a small conical eminence on the body of the parent: this gradually enlarges and becomes cylindrical; a cavity is formed in its interior, which at first is separate, but afterwards comes to communicate with the stomach of the parent, so that aliments taken by the parent penetrate into the stomach of the offspring. As the new polype enlarges, the internal cavity opens at the free extremity, where a mouth, provided with tentacula, is formed. The young animal then catches and swallows food for itself: this food at first finds its way into the stomach of the parent, but after some time all communication between the two stomachs is prevented by the closure of the root of the stem of the small polype; and afterwards the offspring is detached from the parent, becomes a separate individual, and in its turn propagates new ones from its sides. The time at which the separation takes place seems to depend in some measure on the quantity of food within the reach of the parent; this occurring at an early period when the supply is small, and when there may be supposed to be a necessity for the young to move about from place to place in search of sustenance. Sometimes indeed the separation is much retarded, and the young ones also propagate while remaining on the parent stem; so that the polype assumes a branched form, the parent stem bearing families of several generations." 433.

*e. Generation by Separated Buds or Sporules.*—In the last form of non-sexual reproduction the young are formed from small detached masses, after they are separated from the body of the parent. These bodies, generally of a rounded form, may be regarded as buds formed in the parent body, as those of polypes are, but detached from it before the evolution of the new animal begins. They bear the same relation to the offspring as the egg of higher animals to their fœtus or embryo. They are denominated sporæ, germina granulosa, and gemmæ, or germs. They are *supposed* to differ from ova, in being homogeneous in their structure, having no investing membranes, and being entirely converted into the substance of the new animal produced from them.

In some animals these sporules are formed in all parts of the body indiscriminately, and are therefore found dispersed through it; in others there is present a peculiar organ in which they are formed, constituting the simplest form of a reproductive organ. The latter is the more frequent arrangement, and obtains in the greater number of the lower tribes of Mollusca.

*f. Sexual Reproduction.*—This essentially consists in the existence and action of two classes of organs. One, the female, produces the ovum—the other, the male, a fluid which fecundates it.

*g. Hermaphrodite Generation.*—When both kinds of organs exist in the same individual, it is termed hermaphrodite. This arrangement chiefly holds in animals belonging to the Annelida, Acephala, and Gasteropoda.

"In Hermaphrodite animals there are two modes in which fecundation takes place. In some of the Acephala, and in the Holothuriæ, the union of the sexual organs necessary for fecundation takes place in a single individual; while in others, as Helix and Lymneus among the Gasteropoda, copulation, or the union of two individuals, is required, and there is mutual impregnation, the female organ of each animal being fecundated by the male of the other,—a mode of impregnation which also exists in the common Earth-worm, Leech, and some other animals. Occasionally we find that three or more individuals engage in this sort of mutual fecundation, being arranged in a chain or circle." 435.

*h. Diœcious Reproduction.*—When the sexes are distinct, and copulation necessary, the modifications of the generative process hinge chiefly on the mode in which the new animal springs from the egg. When the female parent lays the egg, and the young being is subsequently hatched from it, the mode of reproduction is termed oviparous. When the young are born alive, the generation is called viviparous. Mammalia are generally in the latter category; birds, most reptiles, and fishes in the former.

"In both these classes of animals ova are formed from the ovary, and in both the ova are fecundated within the body of the female parent. The process by which the egg is separated from the place of its formation, and the changes it undergoes in being perfected after its separation, are the same in both: but after the fecundation and completion of the egg, it is differently placed in the two classes of animals; for in birds the egg passes through the oviduct and leaves the body of the female parent, to be hatched into life under the influence of favorable external agents; while in the mammiferous quadruped the egg remains within the uterus of the female generative organs, becomes attached to it, and has there formed from it the young animal which does not quit the body of the parent until it is capable of independent life. The egg of the bird leaves the body of the mother provided with a considerable quantity of organic matter, by which alone, under the influence of heat and air, the embryo is nourished during incubation. The egg of the mammiferous animal is extremely small compared to the size of the young animal at birth, and the fœtus consequently draws a continual supply of the materials of its nourishment from the uterus of the mother, with which it is more or less intimately connected. The residence of the child or young animal in the body of the mother during its formation and growth is termed pregnancy, or utero-gestation." 435.

Some animals bear their young alive, yet the generative organs of the female, as well as the ova themselves, resemble much more closely in their structure those of oviparous than those of viviparous animals. This mode of reproduction consequently gets the name of ovo-viviparous. We do not think it necessary to dwell at greater length upon these points at present. We pass over the consideration of some varieties in respect to uterogestation, and the development of the young, marsupiate generation, monotrematous generation, and a comparison of animal and vegetable reproduction, in order to arrive at the generative function in man and the higher animals. Nor do we think, that, in them, we need dwell on the organs of generation in the respective sexes, on menstruation, puberty, sexual feeling, erection, or so forth, circumstances with which our readers must all be more or less familiar, and on which our author sheds no new light.

3. *Changes in the Female Organs, consequent on Fruitful Sexual Union.*

*a.* The immediate consequence of sexual union is the great excitement of the internal generative organs of the female, and sanguineous turgescence in them. This endures for some time.

*b.* The fimbriated extremites of the Fallopian tubes embrace the ovaria closely.

*c.* The ovarium, unimpregnated, contains the Graafian vesicles. These are filled with fluid coagulable by heat, alkohol, or acids, &c. The membrane forming the vesicle consists of two layers, an external and internal.

In 1827, Baer made the important discovery of the ovum itself, in the fluid contents of the Graafian vesicle.

"Baer found that, in the centre of a granular layer, placed generally towards the most prominent part of the vesicle, to which he gives the name of proligerous disc or layer, there is fixed a very minute spheroid body, seldom above $\frac{1}{200}$th part of an inch in diameter. The appearance of this body he found to be constant, and on examining it with attention in the vesicles of the ovaries, and after their rupture in the Fallopian tubes, he traced the changes that it underwent in the first days after copulation, and established satisfactorily the identity of this body with the ova found by previous observers in the Fallopian tubes and cornua of the uterus; thus proving by actual observation what had before been held only from analogy, that in the mammiferous or truly viviparous, as well as in the oviparous animal, the fœtus derives its origin from an ovum already formed in the ovary before fecundation.

Some time after sexual union the fluid contained in the vesicles which are about to burst, previously transparent and nearly colourless, now becomes more viscid and tenacious, somewhat turbid and of a reddish colour; and in some animals it is possible in such ripe vesicles to perceive, with the unassisted eye in a favourable light, a whitish opaque spot on the most prominent part, indicating the layer of granules or proligerous disc, in the centre of which the ovum is situated. After a certain time a small opening is formed at the most prominent part of the coverings of the vesicle, the vesicle bursts, and its contents escape through the opening; they are received in the infundibulum, which is now applied firmly against the ovary; and the ovum entering the Fallopian tube is conveyed along it, probably by its slow and gradual vermicular contractions, until it at last arrives in the uterus." 449.

*d.* After the Graafian vesicles have burst, important changes occur in them and in the neighbouring ovary.

In the vesicle which is about to burst, the bloodvessels at its most prominent part converge to the point at which the rupture afterwards takes place, the point itself being comparatively deprived of them. When the vesicle is emptied of its fluids, their place is supplied by effused blood.

"The membranes of the vesicle at this time have become thicker than before; the inner one in particular appears more vascular and uneven, perhaps in part from its being puckered up on the vesicle becoming flaccid and comparatively empty. The wrinkled appearance on the inner surface of the vesicle increases, and there grows gradually out from it a new substance which comes to occupy the whole cavity of the vesicle; and in many instances, as this new substance is formed in greater quantity than can be contained within the limits of the vesicle, it protrudes some way out at the opening of the vesicle, forming a dark red prominence like a nipple, which rises above the neighbouring surface of the ovary. This substance, at the time of its first formation, is of a pink or reddish colour, but as it becomes gradually less filled with blood it acquires a yellowish hue, which is more or less apparent in different animals. In the human species it is of a bright yellow colour, whence the name of corpus luteum applied to this new production of the ovarian vesicles. The substance of the corpus luteum has a lobular structure; the lobules radiating in a somewhat irregular manner from the centre to the circumference. The central part of the corpus luteum frequently remains hollow for some time after its production, opening exteriorly by a narrow passage from the place where the rupture of the vesicle originally took place; at other times this passage is closed more early, and there remains nothing but an indication of its place in a depression in the centre of the most projecting part of the corpus luteum. The lobules of the corpus luteum, examined with the microscope, exhibit merely a granular structure, and are not formed of acini, as some have described them, so that there is no reason to consider these bodies as of a glandular nature." 449.

In woman, the corpus luteum attains the size of a hazel nut—afterwards it decreases, and either wholly disappears, or leaves only a small cicatrix. It continues during pregnancy, and diminishes with comparative rapidity after it.

In the sow and mare, venereal excitement causes rupture of the ovarian vesicles, and all the subsequent changes. In the human female, this occasionally happens. Corpora lutea in her are not certain signs of sexual union. But it is only when conception and pregnancy occur that they attain their full dimensions, and run through the whole series of their changes.

The uses of the corpora lutea are unknown.

Here is a natural pause. The changes of the ovum will form a distinct article on a convenient opportunity.

